# Antimicrobial Prescribing Preparedness of Croatian Medical Students—Did It Change between 2015 and 2019?

**DOI:** 10.3390/medicines10070039

**Published:** 2023-06-29

**Authors:** Dora Palčevski, Andrej Belančić, Ivan Mikuličić, Eduard Oštarijaš, Robert Likić, Oliver Dyar, Vera Vlahović-Palčevski

**Affiliations:** 1 Clinical Hospital Centre Rijeka, 51000 Rijeka, Croatia; 2 Department of Clinical Pharmacology, Clinical Hospital Centre Rijeka, 51000 Rijeka, Croatia; veravp@medri.uniri.hr; 3 Faculty of Medicine, University of Rijeka, 51000 Rijeka, Croatia; ivan77mikulicic@gmail.com; 4 Institute for Translational Medicine, University of Pécs Medical School, Szigeti, 7601 Pécs, Hungary; 5 School of Medicine, University of Zagreb, 10000 Zagreb, Croatia; robert.likic@mef.hr; 6 Division of Clinical Pharmacology, University Hospital Centre Zagreb, 10000 Zagreb, Croatia; 7 Department of Public Health and Caring Sciences, Uppsala University, 75237 Uppsala, Sweden; oliver.dyar@pubcare.uu.se; 8 Faculty of Health Studies, University of Rijeka, 51000 Rijeka, Croatia

**Keywords:** antimicrobial prescribing, antimicrobial stewardship, education, medical students, undergraduate medical schools

## Abstract

Background: Antimicrobials are some of the most prescribed drugs by junior doctors, but studies suggest most medical graduates feel unprepared for their future prescribing tasks. The aim of the present study was to compare the self-reported preparedness to prudently prescribe antimicrobials of final-year medical students in Croatia in 2015 and 2019. Methods: The same self-reported web-based survey on the preparedness to prescribe antibiotics was used in both 2015 and 2019. All final-year students at all four medical schools in Croatia (Osijek, Rijeka, Split, and Zagreb) were invited to participate in both 2015 and 2019. Preparedness scores were divided into “topic preparedness scores” and “global preparedness scores”. Topic preparedness scores represented the percentage of students at a medical school who felt sufficiently prepared for each topic. They were first established at a medical school level and then at the national level. Global preparedness scores were determined for each student separately and then calculated at the medical school and national levels. Results: The country’s global preparedness score, representing the average proportion of topics in which students felt sufficiently prepared, was slightly higher in 2015 compared with the 2019 results (62.7% vs. 56.5%; *p* = 0.191). Croatian students reported higher preparedness in 2015 than in 2019 for 25 out of 27 topics included in the survey. The majority of students reported a need for more education on antibiotic use both in 2015 and 2019 (78.0% vs. 83.0%; *p* = 0.199). Conclusions: Despite increasing antimicrobial stewardship activities in various healthcare settings, medical students who are about to start prescribing antibiotics on their own do not feel sufficiently prepared to do so. Antimicrobial stewardship programs should be designed to incorporate undergraduate medical student education, for instance, as a specific, mandatory course or integrated into other courses, such as clinical pharmacology.

## 1. Introduction

According to the European Association of Clinical Pharmacology and Therapeutics, a competent medical graduate should have adequate knowledge of a list of the most frequently prescribed drugs, the ability to successfully treat common medical conditions, a rational approach to drug selection, and the capability to write a prescription safely and unambiguously [1]. However, several studies suggest that most medical graduates feel unprepared for their future prescribing tasks [2,3]. This is a major concern since junior doctors are responsible for a significant proportion of prescriptions and, consequently, make relatively many prescribing errors [4,5].

The complexity of antimicrobial prescribing in daily practice accompanied by serious gaps in prescribing skills and knowledge can contribute to high rates of antimicrobial misuse and overuse [6,7]. Previous studies have suggested that medical students are aware of high antibiotic misuse in clinical practice and its consequences while also feeling individually unprepared for adequate prescribing at least in part due to insufficient education on antimicrobial use [3,6]. For example, Student-PREPARE, a large cross-sectional study that assessed medical students’ self-reported preparedness for responsible antibiotic use in 29 European countries in 2015 showed that many final-year European medical students did not feel sufficiently prepared to responsibly prescribe antibiotics [6]. Interventions to promote prudent antimicrobial prescribing should be increasingly targeted at undergraduates rather than at postgraduates only, as was recently highlighted by the World Health Organization (WHO) in the Global Action Plan on Antimicrobial Resistance [8]. Antimicrobial stewardship targeting undergraduate medical students is likely to be effective since the knowledge, attitudes, and behaviors of future prescribers are still being formed while they are in medical school [9].

### Antibiotic Stewardship in Croatia

In Croatia, the Interdisciplinary Section for Antimicrobial Resistance Control (ISKRA) (www.iskra.bfm.hr (accessed on 1 May 2023)) coordinates various national antimicrobial stewardship activities such as antibiotic consumption and resistance surveillance, the development of antibiotic prescribing guidelines, awareness campaigns targeted at lay public and professionals, as well as occasional educational seminars and symposia [10]. To the best of our knowledge, such activities have not been specifically designed and targeted at final-year medical, dental, or pharmacy students in Croatia.

In Croatian medical schools, most of the knowledge about antibiotics and their appropriate use is taught during microbiology and basic pharmacology courses in the third year and a year later in infectious disease placements [11]. The total lengths of the medical programs in Croatian medical schools are six years.

Studies conducted at the national level in Croatia show a slightly lower rate of adherence to antimicrobial guidelines (compared with the European average), a general tendency/ease to prescribe antimicrobials without a clear indication among Croatian physicians, and unfavorable antimicrobial resistance rates [10,12,13,14]. Antimicrobial consumption and bacterial resistance rates in Croatia are higher than the European average according to European Center for Disease Prevention and Control Annual Epidemiological Reports on antimicrobial consumption and resistance rates [15,16].

The aim of the present study was to compare the self-reported preparedness for a comprehensive set of topics related to prudent antimicrobial use between final-year medical students in Croatia that were included in the Student-PREPARE study (2015 yr. graduates) and a subsequent survey of 2019 yr. graduates. In general, there were no major differences in the development or usage of teaching methods between 2015 and 2019, and there were no specific changes to the curriculum between the two time points. However, there have been more general efforts in clinical settings in recent years to raise the importance of prudent antibiotic prescribing.

An additional aim of this study was to assess whether general antimicrobial stewardship efforts in recent years have affected final-year medical students’ preparedness for specific curriculum topics, aiming to lay the basis for a reform of medical curricula and closer integration of stewardship activities in undergraduate education for medical students. We hypothesized that despite the increase in antimicrobial stewardship activities in various healthcare settings in general, medical students still do not feel sufficiently prepared to prescribe antibiotics on their own and desire more education on this topic.

## 2. Materials and Methods

### 2.1. Study Design and Participants

In 2015, a cross-sectional, multicenter, closed web-based survey was designed by members of the European Study Group on Antimicrobial Stewardship (ESGAP) Student-PREPARE Working Group and disseminated among medical schools in 29 European countries in order to assess medical students’ self-reported preparedness for prudent antibiotic use [6]. The same survey was used again in 2019 in Croatia, and all final-year students at all four medical schools in Croatia (Osijek, Rijeka, Split, and Zagreb) were invited to participate. There were no additional exclusion criteria. The self-administered survey was accessible on SurveyMonkey^®^ from 20 January until 1 July 2015, and from 11 May until 1 July 2019. In 2015 and 2019, the survey was advertised to students via an email from a student representative at each medical school, followed by up to two email reminders at schools with low response rates. Eligible students at participating schools were sent invitations to a Facebook student group by local student coordinators. After a few weeks, additional reminders were sent to schools with low response rates. The ability to submit multiple responses from the same device was disabled in SurveyMonkey. Participation was voluntary, anonymous, and without compensation and, as such, did not require ethics committee approval.

### 2.2. Survey Development

The survey tool was developed by a committee of international experts on antibiotic stewardship and was influenced by previous studies on the effects of undergraduate curricula on medical students [6]. Its complete design and development have been comprehensively described elsewhere by Dyar et al. [6]. The 47-item questionnaire included questions on demographics, self-reported preparedness for 27 curricular topics on prudent antibiotic use on a 7-point Likert-type scale, perceptions of the usefulness of teaching methods, and the perceived need for further education (available at https://doi.org/10.1093/jac/dky150 (accessed on 1 May 2023)) [6]. The survey was not translated into Croatian and could only be completed in English. The survey items were not randomized but instead grouped around related topics. There was a maximum of 3 questions per page, some of which had 3–8 sub-questions. The survey consisted of 13 pages, and it was estimated that it needed 5–10 min to be completed. The respondents were able to review their responses by clicking the ‘Back’ button.

### 2.3. Statistical Analyses

Answers to questions on preparedness concerning the 27 curricular topics were divided into 2 categories (4–7: at least sufficiently prepared; 1–3: insufficiently prepared).

Preparedness scores were categorized as “topic preparedness scores” and “global preparedness scores”. Topic preparedness scores represented the percentage of students at a medical school who felt sufficiently prepared for each topic. They were first determined at the medical school level and then at the national level. Global preparedness scores, representing the percentage of the 27 topics for which students felt at least sufficiently prepared, were calculated for each student separately and then aggregated at the medical school level and then the country level.

Data were exported from the SurveyMonkey^®^ service, and statistical analysis was performed using Microsoft Excel 2016^®^ (Microsoft Office) and MedCalc v12.1.3 (MedCalc Software bvba, Ostend, Belgium). Responses in which fewer than half of the questions were answered were not taken into consideration.

Comparisons between the availability of teaching methods according to the year of graduation, the perceived needs for further education, and preparedness scores were conducted using a chi-square test. The criterion for statistical significance was estimated at *p* < 0.05.

## 3. Results

### 3.1. Participants

We received a total of 318 responses in 2015 and 159 in 2019 from the four medical schools in Croatia: Osijek, Rijeka, Split, and Zagreb. The overall response rates for 2015 yr. graduates and 2019 yr. graduates were 59.1% (N = 318) and 29.6% (N = 159), respectively (*p* < 0.001). No significant difference in the gender distribution was observed when comparing the 2015 yr. and 2019 yr. graduates (66.4% female in 2015 vs. 65.9% female in 2019; *p* = 0.919). The mean age was similar in both year groups (24.4 vs. 24.8). Of all collected responses, 2.2% of the 2015 graduates and 1.3% of the 2019 graduates would have preferred the survey to have been in the native (Croatian) language (*p* = 0.477).

### 3.2. Global Preparedness Scores

The country’s global preparedness score, representing the percentage of topics for which students felt at least sufficiently prepared, was similar in 2015 and 2019 (62.7% in 2015 vs. 56.5% in 2019; *p* = 0.191). The medical school global preparedness scores ranged from 55.6 to 67.9% in 2015 and from 48.8 to 66.3% in 2019. The medical school global preparedness scores were highest in Split and lowest in Osijek for both studied years.

### 3.3. Preparedness for Individual Curriculum Topics

Croatian students reported higher preparedness in 2015 than in 2019 for 25 of the 27 topics included in the survey, with 16 of these differences being statistically significant. The only questions for which students reported being more prepared in 2019 (both non-significant) were “To practice effective Infection control and hygiene (to prevent spread of bacteria)” and “To use knowledge of the negative consequences of antibiotic use (bacterial resistance, toxic/adverse effects, cost, Clostridium difficile infections)”. The estimated percentages of students who were prepared for each individual topic in both 2015 and 2019 are presented in Table 1.

The topic that students reported being most prepared for was “To recognize the clinical signs of infection” (93.7% vs. 88.7%; *p* = 0.056) in both years, followed by preparedness “To interpret biochemical markers of inflammation” (89.6% vs. 88.1%; *p* = 0.760) and “To differentiate between bacterial and viral upper respiratory tract infections” (89.0% vs. 85.5%; *p* = 0.191).

The topic for which students felt the least prepared was “To measure/audit antibiotic use in a clinical setting, and to interpret the results of such studies” in both 2015 and 2019 (41.2% vs. 27. 7%; *p* = 0.004).

The ranking of topics was similar in both 2015 and 2019, especially at the top of the list. Seventy percent of topics were within three places of their ranking in both years. More differences were noticed in the ranking of the topics for which the students felt less prepared.

In addition to the 7-point Likert scale, participants could also answer that no teaching was provided for a certain topic. The questions that were most frequently reported as not being taught were the same in both years, and they included “To measure/audit antibiotic use in a clinical setting, and to interpret the results of such studies” (6.6% vs. 16.4%; *p* = 0.004), “To work within the multidisciplinary team in managing antibiotic use in hospitals”(6.9% vs. 17.0%; *p* = 0.002), and “To communicate with senior doctors in situations where I feel antibiotics are not necessary, but I feel I am being inappropriately pressured into prescribing antibiotics by senior doctors” (6.0% vs. 10.7%; *p* = 0.003).

**Figure 1:**
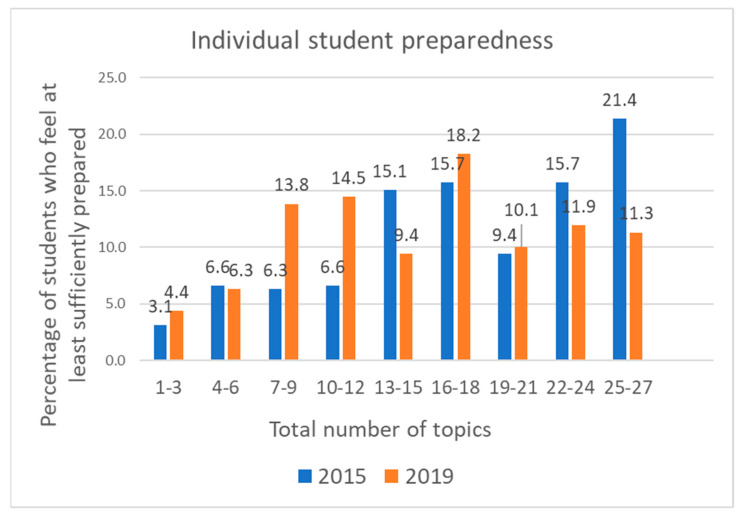
Distribution of total number of topics in which individual students felt at least sufficiently prepared.

The percentage of students who felt sufficiently prepared for more than 89% (24 out of 27 questions) of the topics was almost twice as high in 2015 than in 2019 (Figure 1).

### 3.4. Perceptions of Antimicrobial Education

**Figure 2:**
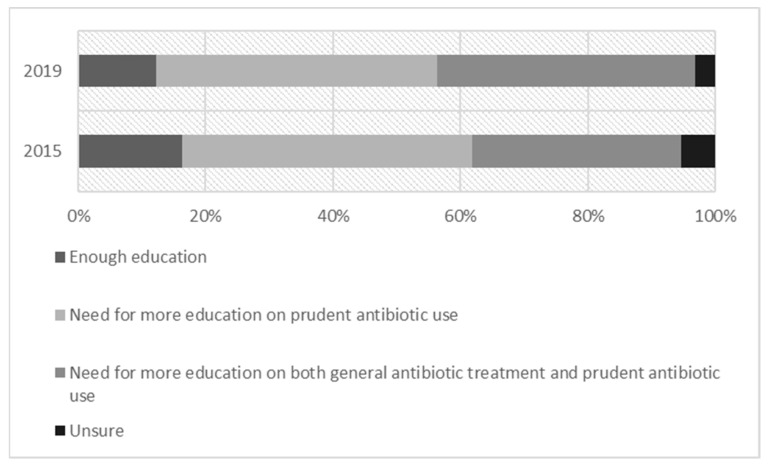
Expressed need for more education.

The majority of surveyed students in both years reported feeling the need for additional education on antibiotic use for their future work (78.0% vs. 83.0%; *p* = 0.199) (see Figure 2). The results did not vary significantly between the four universities in 2015 (72.2–81.4%) or in 2019 (73.7–100%).

Students in 2019 provided suggestions more frequently to the open-style question “How do you think teaching on antibiotic treatment and prudent antibiotic use can be improved?” (65.4% vs. 27.4%; *p* < 0.001). In both years, the most frequently mentioned groups of answers were *more case presentations* (9.8% in 2015 vs. 25.2% in 2019; *p* < 0.001), *including students in clinical practice* (7.2% in 2015 vs. 20.8% in 2019; *p* < 0.001), *more specific epidemiological teaching in the form of local guidelines* (5.0% in 2015 vs. 6.9% in 2019; *p* = 0.400), and *more attention to the topic during medical undergraduate studies.* A specific problem some students mentioned was that after the infectious diseases placement in the fourth year, the topic of antimicrobial stewardship is not mentioned as much in later courses, so students do not remember the content sufficiently well when they start working three years later.

### 3.5. Teaching Methods

In both 2015 and 2019, the prevailing teaching method used for the subject of antibiotic prescribing was *lectures for more than 15 students*, and the least utilized method was *role-play or communication skills sessions dealing with patients demanding antibiotic therapy*. The perceived availability and usefulness of different teaching methods for antibiotic use are presented in Table 2. Statistically significant differences were found for the following teaching methods between 2015 and 2019: *discussion of clinical cases and vignettes* (*p* = 0.022), *active learning assignments* (*p* < 0.001), and *role-play or communication skills* (*p* < 0.001).

## 4. Discussion

### 4.1. Overall Preparedness

Our study evaluated self-reported preparedness among final-year medical students at Croatian medical schools using an identical questionnaire in both 2015 and 2019. Students reported higher preparedness levels for almost all individual topics in 2015, although the overall percentage of topics for which students felt at least sufficiently prepared was similar between the two years.

One of the possible reasons for students feeling slightly more prepared in 2015 might be due to the cohort of 2019 being the first generation of Croatian students that would obtain a medical license without having to undergo a mandatory five-month-long internship. The survey was conducted at the end of the 2019 academic year when the surveyed students did not know whether or not they would start working without any supervision in a few more months because, at that point in time, the subject still had not been decided at the national level. That may have led to a general feeling of uncertainty and a lack of confidence that might have affected the results and left the students feeling insecure about their knowledge [17,18]. Students may have also perceived that a greater amount of knowledge is required for residency (in 2019) than for an internship (in 2015). A similar pattern of results was observed in studies in France (where students enter directly into specialty training) and Sweden (where students enter an internship after their studies), wherein students in France felt less prepared than those in Sweden [19].

A major public campaign for rational antibiotic use was carried out from 2009 to 2015, so the final-year medical students in 2015 may have been more exposed to the topic and could have recognized prudent antibiotic use as more important. Among the students of 2019, the topic might have been perceived as being less relevant, as it was not mentioned as often during their studies. This suggests that recurrent reminders on prudent antibiotic use are required.

The explanation that graduates of Generation 2015 might have gained a better knowledge of antimicrobial prescribing is quite unlikely, given that neither the curricula nor the teaching methods had changed during that period. Although some antibiotic stewardship activities have been introduced at the national level since 2015, medical students have not been the targeted audience.

When comparing our results from both 2015 and 2019 with those in other European countries that used the same survey, the Croatian global preparedness scores in 2019 were similar to the European average [6], but they were considerably lower than the results in some countries such as Latvia (85.1%), Finland (84.3%), and Sweden (83.4%). The very high percentage of students who both stated they would prefer more education on the topic and further offered suggestions for improvement in education on the topic indicates that Croatian students are aware of the problem. Similar awareness among students of the problem has been seen in previous studies [2,3].

### 4.2. Preparedness for Individual Topics

Although preparedness levels were lower for almost all topics in 2019, there were very few marked variations for individual topics between years, which is in keeping with the lack of changes in undergraduate education during these years. Together with the similarity in the ranking of the topics between the two studies, these findings suggest that the variations in preparedness between topics may be either due to certain topics being inherently more difficult to feel prepared for or secondary to a continuous deficit in training on specific topics, which was present at both time points. Furthermore, the three topics for which Croatian students reported the highest levels of “no provided teaching” were the same as in several other European countries [6,19], suggesting that these topics were generally not taught enough during medical education. These topics were “To measure/audit antibiotic use in a clinical setting, and to interpret the results of such studies”, “To work within the multidisciplinary team in managing antibiotic use in hospitals”, and “To communicate with senior doctors in situations where I feel antibiotics are not necessary, but I feel I am being inappropriately pressured into prescribing antibiotics by senior doctors”. In contrast with the summary statistics on preparedness for individual topics, which were similar in both years, greater differences were seen in the levels of preparedness at the individual student level. The percentage of students who felt sufficiently prepared for 90% of topics was considerably higher in 2015.

Our surveys were conducted at the end of the final year of medical school. The fact that the majority of students still expressed a need for more education indicates that additional efforts are needed to emphasize antimicrobial stewardship during undergraduate and postgraduate medical education. The perceived need for more education was expressed to a greater extent among Croatian medical students than it was among students in many other European countries (e.g., 63.5% in France and 20.3% in Sweden) [19]. Supporting these findings, 45% of students in 2019 answered the open-style question with specific suggestions for how education could be improved and emphasized the need for more teaching focused on providing specific guidelines.

### 4.3. Teaching Methods

Since Croatian medical curricula use all teaching methods, the lower preparedness scores may have been caused by poor implementation of materials and methods, rather than by their complete absence. This was demonstrated in a recent study by Tim van der Voort et al., who found that students prescribed antibiotics with much greater accuracy in countries that used problem-solving teaching methods [20].

In addition, education could employ peer-to-peer teaching more frequently. This was one of the least utilized methods, but 77.3% of students responding in 2019 who had been exposed to peer-to-peer teaching found it useful or very useful in this particular subject context. Another tool that could be useful for teaching students and young doctors about AMS is the availability of specific guidelines that are used in everyday practice. Students should be given their own copies, and they should be taught how to use them in real clinical settings. Similar to other studies, a frequently mentioned suggestion in the open-style questions (by approximately one-fourth of the students in 2019) was to include students in the clinical practice, exposing them to the responsibility of prescribing antibiotics during their education under the guidance of supervisors [21]. Involving students in public antibiotic awareness campaigns could also be an educational and motivating factor to underline the importance of the antibiotic resistance problem and empower students to better understand its relevance.

Finally, it would certainly help students to both feel and be more prepared to prescribe antibiotics responsibly if this topic were to feature not only in the fourth year but also later in the medical program, particularly given that antibiotics are among the most common drugs that medical students will prescribe after graduation. This could be, for example, in the context of the course *Clinical Pharmacology* that students undertake in the last year of their studies, which deserves more attention and ECTS points [11,22]. As the students’ responses suggest, such training towards the end of the medical program could have a strong focus on developing practical skills in antimicrobial prescribing to complement earlier more theoretical teaching.

Finally, since knowledge and skills on the rational prescribing of antimicrobials are further refined through subsequent clinical practice as a junior doctor, it is worth considering implementing stronger support for early postgraduates, e.g., enhanced feedback through mentors or multidisciplinary teams.

### 4.4. Strengths and Limitations

This was the first survey conducted in Croatia to assess preparedness for antimicrobial prescribing among final-year medical students, and it included respondents from all medical schools, so the results should be broadly representative of all final-year medical students in Croatia. The questionnaire was anonymous, reducing the impact of social biases. Our study also had some limitations. The response rate in 2019 was much lower than in 2015. However, the rate in 2019 (slightly under 30%) was similar to those obtained in other European countries the same survey has been conducted [9] as well as in a study in the US [23]. Importantly, the 2015 pan-European study found no evidence of a correlation between the response rates at individual medical schools and preparedness levels. A potential limitation for interpreting some of our results is the lack of power calculation, i.e., the study may not have been sufficiently powered to detect small differences in preparedness levels (<5% between comparison years). We chose not to conduct such power calculations since it would not affect our participant selection strategy; the inclusion criterium consisted of all final-year medical students studying at a Croatian medical school rather than a sampling of a subset. Additionally, the original questionnaire was conducted in English. Although only a small number of students reported that they would have preferred the survey to be in Croatian, the foreign language might have affected their understanding of the questions and the results, but it should have had a similar effect on the results for 2015 and 2019. Finally, a comparison between different medical schools in Croatia was not the objective of this study, so we did not perform such analyses. Importantly, such comparisons would likely be highly underpowered. The quality of education on antimicrobial preparedness may vary between medical schools, which could influence the presentation of aggregated results in case of a different distribution of participants per medical school (i.e., between 2015 vs. 2019 yr.); however, this was not the case in our study since the distribution per site was relatively unchanged between the study years.

## 5. Conclusions

Despite increasing antimicrobial stewardship activities in various healthcare settings, final-year medical students in Croatia who are about to start prescribing antibiotics independently do not feel sufficiently prepared to do so. Antimicrobial stewardship programs should be designed to specifically target undergraduate students. In conclusion, the obtained results should form the basis for tailoring and updating Croatian medical curricula as well as ongoing stewardship activities. Finally, it would be interesting to explore the impact of the COVID-19 pandemic on students’ self-reported preparedness for prudent antimicrobial use since educational methods and content have changed significantly, and students may have been exposed to more liberal antibiotic use policies in clinical practice.

## Figures and Tables

**Table 1:** Self-reported preparedness levels for prudent antibiotic use.

Topic		Sufficiently Prepared		*p*	Trend
		2015 (%), N = 318	2019 (%), N = 159		
1	To recognize the clinical signs of infection	93.7	88.7	0.056	=
2	To differentiate between bacterial and viral upper respiratory tract infections	89.6	85.5	0.191	=
3	To interpret biochemical markers of inflammation (e.g., CRP)	89.0	88.1	0.76	=
4	To practise effective infection control and hygiene (to prevent spread of bacteria)	83.7	84.9	0.723	=
5	To interpret basic microbiological investigations (e.g., blood cultures, antibiotic susceptibility reporting)	81.8	81.1	0.867	=
6	To use knowledge of the negative consequences of antibiotic use (bacterial resistance, toxic/adverse effects, cost, Clostridium difficile infections)	81.8	84.9	0.391	=
7	To identify clinical situations when not to prescribe an antibiotic	80.8	68.6	0.003	↘
8	To discuss antibiotic use with patients who are asking for antibiotics, when I feel they are not necessary	79.9	70.4	0.022	↘
9	To differentiate between bacterial colonisation and infection (e.g., asymptomatic bacteriuria)	78.6	64.8	0.001	↘
10	To assess the clinical severity of infection (e.g., using criteria, such as the septic shock criteria)	78.3	72.3	0.149	=
11	To decide when it is important to take microbiological samples before starting antibiotic therapy	73.9	64.8	0.039	↘
12	To use knowledge of the common mechanisms of antibiotic resistance in pathogens	68.6	61.6	0.133	=
13	To assess antibiotic allergies (e.g., differentiating between anaphylaxis and hypersensitivity)	61.3	50.9	0.031	↘
14	To prescribe antibiotic therapy according to national/local guidelines	59.1	48.4	0.027	↘
15	To assess clinical outcomes and possible reasons for failure of antibiotic treatment	57.6	44.0	0.005	↘
16	To use point-of-care tests (e.g., urine dipstick, rapid diagnostic tests for streptococcal pharyngitis)	57.2	43.4	0.004	↘
17	To use knowledge of the epidemiology of bacterial resistance, including local/regional variations	55.4	41.5	0.004	↘
18	To decide the urgency of antibiotic administration in different situations (e.g., <1 h for severe sepsis, non-urgent for chronic bone infections)	54.7	42.8	0.014	↘
19	To review the need to continue or change antibiotic therapy after 48–72 h, based on clinical evolution and laboratory results	54.4	49.7	0.332	=
20	To select initial empirical therapy based on the most likely pathogen(s) and antibiotic resistance patterns, without using guidelines	53.8	44.7	0.061	=
21	To communicate with senior doctors in situations where I feel antibiotics are not necessary, but I feel I am being inappropriately pressured into prescribing antibiotics by senior doctors	49.7	35.2	0.003	↘
22	To identify indications for combination antibiotic therapy	44.0	27.7	0.001	↘
23	To work within the multi-disciplinary team in managing antibiotic use in hospitals	44.0	28.9	0.002	↘
24	To decide when to switch from intravenous (IV) to oral antibiotic therapy	43.4	28.9	0.002	↘
25	To prescribe using principles of surgical antibiotic prophylaxis	42.5	32.7	0.04	↘
26	To decide the shortest possible adequate duration of antibiotic therapy for a specific infection	42.1	37.1	0.292	=
27	To measure/audit antibiotic use in a clinical setting, and to interpret the results of such studies	41.2	27.7	0.004	↘

= a change that is not significant; ↘ a significant decrease.

**Table 2:** Availability and usefulness of different teaching methods.

Teaching Method	2015 (N = 318)			2019 (N = 159)		
	Useful or Very Useful (%)	Not Very Useful (%)	Not Available (%)	Useful or Very Useful (%)	Not Very Useful (%)	Not Available (%)
Lectures (with > 15 people)	53.5	10.7	0.3	49.7	12.6	1.5
Small group teaching (with < 15 people)	77.4	0.6	6.9	71.7	3.8	13.2
Discussions of clinical cases and vignettes	79.3	1.3	8.8	69.8	1.9	12.6
Active learning assignments (e.g., article reading, group work, preparing an oral presentation)	45.9	7.2	15.4	25.8	19.5	21.4
E-learning	19.2	11.0	41.5	23.9	8.2	44.0
Role play or communication skills sessions dealing with patients demanding antibiotic therapy	35.9	3.5	42.1	20.1	6.3	52.2
Infectious diseases clinical placement (i.e., clinical rotation or training in infectious diseases, involving patients)	66.3	2.8	11.6	66.0	4.4	7.6
Microbiology clinical placement	46.5	7.9	12.0	43.4	10.7	10.0
Peer or near peer teaching (i.e., teaching led by other students, or recently qualified doctors)	43.7	8.2	18.9	36.5	10.7	32.1

* In the survey, two more categories were available: “Neutral” and “I am unsure”. ** “Not available” means that the teaching method was not used at a specific university.

## Data Availability

The data are available upon request.
